# Abnormal resting-state functional connectome in methamphetamine-dependent patients and its application in machine-learning-based classification

**DOI:** 10.3389/fnins.2022.1014539

**Published:** 2022-11-17

**Authors:** Yadi Li, Ping Cheng, Liang Liang, Haibo Dong, Huifen Liu, Wenwen Shen, Wenhua Zhou

**Affiliations:** ^1^Department of Radiology, Ningbo Medical Treatment Center Lihuili Hospital, Ningbo University, Ningbo, China; ^2^Department of Academic Research, Ningbo Kangning Hospital, Ningbo University, Ningbo, China; ^3^Key Laboratory of Addiction Research of Zhejiang Province, Ningbo, China

**Keywords:** methamphetamine, resting-state, connectome, graph theory, machine-learning

## Abstract

Brain resting-state functional connectivity (rsFC) has been widely analyzed in substance use disorders (SUDs), including methamphetamine (MA) dependence. Most of these studies utilized Pearson correlation analysis to assess rsFC, which cannot determine whether two brain regions are connected by direct or indirect pathways. Moreover, few studies have reported the application of rsFC-based graph theory in MA dependence. We evaluated alterations in Tikhonov regularization-based rsFC and rsFC-based topological attributes in 46 MA-dependent patients, as well as the correlations between topological attributes and clinical variables. Moreover, the topological attributes selected by least absolute shrinkage and selection operator (LASSO) were used to construct a support vector machine (SVM)-based classifier for MA dependence. The MA group presented a subnetwork with increased rsFC, indicating overactivation of the reward circuit that makes patients very sensitive to drug-related visual cues, and a subnetwork with decreased rsFC suggesting aberrant synchronized spontaneous activity in subregions within the orbitofrontal cortex (OFC) system. The MA group demonstrated a significantly decreased area under the curve (AUC) for the clustering coefficient (Cp) (*P*_perm_ < 0.001), shortest path length (Lp) (*P*_perm_ = 0.007), modularity (*P*_perm_ = 0.006), and small-worldness (σ, *P*_perm_ = 0.004), as well as an increased AUC for global efficiency (E.glob) (*P*_perm_ = 0.009), network strength (Sp) (*P*_perm_ = 0.009), and small-worldness (ω, *P*_perm_ < 0.001), implying a shift toward random networks. MA-related increased nodal efficiency (E.nodal) and altered betweenness centrality were also discovered in several brain regions. The AUC for ω was significantly positively associated with psychiatric symptoms. An SVM classifier trained by 36 features selected by LASSO from all topological attributes achieved excellent performance, cross-validated prediction area under the receiver operating characteristics curve, accuracy, sensitivity, specificity, and kappa of 99.03 ± 1.79, 94.00 ± 5.78, 93.46 ± 8.82, 94.52 ± 8.11, and 87.99 ± 11.57%, respectively (*P*_perm_ < 0.001), indicating that rsFC-based topological attributes can provide promising features for constructing a high-efficacy classifier for MA dependence.

## Introduction

According to the World Drug Report 2021^1^ and Annual Report on Drug Control in China in 2021,^2^ approximately 3.59 million people worldwide use drugs each year, and methamphetamine (MA) is the most abused drug in China and one of the most abused across the world. Long-term use of MA causes molecular alterations in the dopamine system, contributing to nerve terminal impairment in the central nervous system and leading to damaged motor skills, rapid cognitive decline, increased anxiety, psychotic disorders, violent behavior, hallucinations, delusions, and depression (Rusyniak, 2013).

In recent years, functional connectivity (FC) has gained visibility as a salient tool for assessing functional brain organization and as an important biomarker for neurological disorders (Pervaiz et al., 2020). The resting-state-based functional connectome has been extensively applied to help reveal neurological mechanisms, as well as applied for diagnosis and prediction of treatment outcomes, in patients with substance use disorders (SUDs), such as cannabis (Ramaekers et al., 2022), heroin (Sun et al., 2018), cocaine (Yip et al., 2019), and MA dependence (Kohno et al., 2016; Ipser et al., 2018; Malina et al., 2021). Kohno et al. (2016) analyzed resting-state FC (rsFC) between brain networks and reported that acute exposure to MA increased connectivity between both the thalamus and cerebellum to sensorimotor areas and the middle temporal gyrus and decreased connectivity between the sensorimotor and middle temporal gyrus networks. In terms of chronic MA dependence, Ipser et al. (2018) found an increased correlation between anterior and posterior default mode networks (DMN), which became less apparent with increasing duration of abstinence from MA. By combining ^18^F-fallypride positron emission tomography with rsFC studies in MA-dependent patients, Kohno et al. (2016) concluded that ventral striatal D2-type receptor signaling may affect the system-level activity within the mesocorticolimbic system, providing a functional link that may help explain high impulsivity in patients.

To date, there is no single widely accepted standard for estimating FC. A full (Pearson) correlation analysis between brain regions of interest (ROIs) or voxels is the most commonly used method to analyze FC; however, this approach cannot distinguish whether two brain regions are connected through direct or indirect pathways (Pervaiz et al., 2020). To alleviate this drawback, Pervaiz et al. (2020) defined partial correlations as the correlations between the time series of two network nodes after adjusting for the time series of all other network nodes. This method, however, becomes problematic when there are not considerably more timepoints than nodes. The Tikhonov regularization, one of the approaches based on regularization, also referred to as L2 ridge regression involving a regularization term Γ = αI, was implemented to address these problems. As an efficient and stable method, Tikhonov regularization has been shown to increase predictive power compared to simple partial correlations and hence outperformed other connectivity estimation techniques in terms of both qualitative and quantitative evaluation (Pervaiz et al., 2020).

As rsFC analyses focus only on relationships between brain regions, graph theory offers a powerful mathematical framework for modeling the human brain as a complex network or graph whose topological architectures can be quantitatively characterized (Xia and He, 2017). This technique has been applied to analyze human brain structural and functional networks in the context of SUDs (Sjoerds et al., 2017; Sun et al., 2017; Pandria et al., 2018). However, few studies have reported the application of rsFC-based graph theory in the context of MA dependence. By comparing 17 MA-dependent individuals with normal controls, Mansoory et al. (2017) reported disrupted global topological graph properties under several network sparsity thresholds (up to 9 out of 41 thresholds). To avoid issues caused by evaluating topological features dependent on an arbitrarily chosen threshold, some studies integrated the curve of the graph indices over a range of thresholds, i.e., the area under the curve (AUC) for each topological attribute, before conducting statistical analysis of network topology attributes. The integrated AUC metric has been used in previous brain network studies and is sensitive for detecting topological alterations in brain disorders (Zhang J. et al., 2011; Zhu et al., 2016; Vriend et al., 2018). In the present study, we hypothesized that MA dependence disrupts rsFC and rsFC-based topological organization of intrinsic functional brain networks. To verify our hypothesis, we collected Tikhonov regularization-based rsFC data from MA-dependent patients and healthy control (HC) subjects and analyzed the AUC for topological attributes of their resting-state functional networks using graph theoretical approaches. Between-group differences and relationships with clinical variables were investigated with univariate methods.

An important emerging trend in the analysis of SUDs brain imaging data is the application of supervised machine-learning techniques on complex, high-dimensional datasets to make predictions at the individual level with high accuracy, making them potential clinically actionable diagnostic/prognostic tools (Barenholtz et al., 2020). To date, the most commonly used machine-learning algorithm in the classification of SUDs [e.g., cannabinoid (Celik et al., 2020), nicotine (Wetherill et al., 2019), cocaine (Mete et al., 2016), and heroin (Zhang Y. et al., 2011) dependence] is the support vector machine (SVM) (Noble, 2006), which can help achieve a good separation of a sample into two groups by determining the hyperplane separating the multidimensional (multivariate) feature spaces of the two classes. Yan et al. (2021) constructed an SVM with rsFC network topological attributes from MA-dependent patients and achieved a classification accuracy of 73.2%. Li et al. (2019) trained an SVM on arterial spin labeling (ASL) data, a variant form of functional magnetic resonance imaging (fMRI), to classify MA-dependent subjects with an accuracy of 89%. By exploiting AUC data from rsFC network topological metrics derived from Tikhonov regularization-based rsFC data, the present study also aimed to develop an SVM that could improve the classification efficacy of MA-dependent individuals.

## Materials and methods

### Participants

Forty-six right-handed, male MA-dependent patients were recruited from the voluntary detoxification ward of Key Laboratory of Addiction Research of Zhejiang Province, which unit has now been incorporated into Ningbo Kangning Hospital since 2020. Forty-six age- and education-matched, right-handed, healthy male subjects were recruited as HCs from local communities. All patients were diagnosed by one expertized psychiatrist. Two expertized psychiatrists interviewed all subjects and collected their clinical data.

The inclusion criteria for MA dependence were (a) meeting the Diagnostic and Statistical Manual of Mental Disorders, Fourth edition, Text revision (DSM-IV-TR) criteria for current MA dependence. All patients received an MRI scan within 4–7 days after the last use of MA. (b) Patients had no current or history of dependence on other drugs of abuse (except nicotine). The exclusion criteria included (a) having a history of psychiatric illness, neurological disorder, or major chronic medical illnesses before MA use and (b) having metallic or electronic devices or implants.

The same inclusion and exclusion criteria were applied for the HCs but without a history of drug abuse or dependence, other than nicotine.

The psychiatric symptoms of MA-dependent patients were rated using the Hamilton Anxiety Rating Scale (HAMA) (Hamilton, 1969) and Brief Psychiatric Rating Scale (BPRS) (Overall and Gorham, 1962). The BPRS covers five factors: anxiety-depression, lack of vitality, activity, hostility-suspicion, and thinking disorder.

This study was approved by the Institutional Review Board of Ningbo Medical Center Lihuili Hospital, Ningbo University, Zhejiang, China. Written informed consent was obtained from all subjects or their relatives.

### Magnetic resonance imaging data acquisition

All MRI data were acquired using a 3.0-T clinical MR image unit (Discovery MR750, GE Healthcare, Milwaukee, WI, USA) with an eight−channel head coil. Conventional axial T2−weighted images had previously been obtained to rule out cerebral infarction or other evident lesions. The structural MRI data were collected using a sagittal T1-weighted three-dimensional sequence [repetition time (TR), 7.4 ms; echo time (TE), 3.2 ms; inversion time, 450 ms; flip angle, 12°; field of view, 25.6 mm × 25.6 mm; matrix, 256 × 256; and voxel size = 1 mm × 1 mm × 1 mm].

Resting-state fMRI (RS-fMRI) data were acquired for 6 min and 40 s using a T2*-weighted gradient-echo planar imaging (EPI) sequence (TR = 2,000 ms; TE = 30 ms; field of view = 24 mm × 24 mm; matrix size = 64 × 64; voxel size = 3.75 mm × 3.75 mm × 4 mm; flip angle = 90°; 38 transverse slices; and 200 phases). All subjects were placed in the supine position with foam padding between their head and the head coil to minimize head motion and instructed to keep their eyes closed and not fall asleep.

### Preprocessing of resting-state-functional magnetic resonance imaging data

The RS-fMRI data were preprocessed using Data Processing Assistant for Resting-State fMRI (DPARSF) toolbox, a convenient plug-in software within DPABI (v6.1_220101) software (Yan et al., 2016). For RS-fMRI data preprocessing, the first four volumes were discarded to avoid signal instability. Slice timing was conducted to compensate for systematic slice-dependent time shifts, and realignment to the first volume was performed to correct for head movement artifacts. The realigned images were spatially transformed to a standard Montreal Neurological Institute template using Advanced Normalization Tools (ANTs) (Avants et al., 2009) (v2.1.0) and resampled to a voxel size of 2 mm × 2 mm × 2 mm. Then, the cerebrospinal fluid signal, white matter signal and 24 head motion parameters were regressed out to produce a residual blood oxygen level-dependent (BOLD) signal. Afterward, the images were smoothed with a 6-mm full-width at half-maximum (FWHM) kernel and bandpass filtered (0.01–0.08 Hz) to reduce the low-frequency drift and physiological high-frequency noise, including the breath and heartbeats. The RS-fMRI data for each subject were checked for head motion. In accordance with the criteria that the translation and rotation of head motion in any direction were not more than 2 mm or 2°, no subjects were disqualified.

### Graph construction and calculation of topological attributes

In brain rsFC networks, graphs are composed of nodes, i.e., brain regions and edges between pairs of nodes. All the graphs in this study were undirected and weighted.

In the present study, a brainnetome atlas was utilized to parcellate the brain into 246 ROIs (105 cortical regions and 18 subcortical regions per hemisphere) as network nodes (Fan et al., 2016). DPABINet, a module within DPABI, was used to quantify the Tikhonov regularization-based correlation, i.e., edge, between the averaged time series of the BOLD signals for each pair of nodes, and then a 246 × 246 connectivity matrix was generated, which was subsequently converted to a normal distribution using Fisher’s r-to-z transformation. To exclude possible effects of spurious correlations between network nodes, a sparsity threshold (i.e., the ratio of the number of existing edges divided by the maximum possible number of edges in a network) was applied to individual connectivity matrices to retain only high correlations. As there is currently no definitive way to select a single threshold, each connectivity matrix was empirically thresholded using a wide range of sparsity thresholds [0.05, 0.4] (interval = 0.01) to generate sparse and weighted networks.

A number of topological attributes of the rsFC networks were calculated for each subject under every sparsity threshold using the brainGraph package^3^ in R (v4.1.0) for Linux (R Core Team, 2018). The global-level attributes included clustering coefficient (Cp), shortest path length (Lp), network strength (Sp), local efficiency (E.loc), global efficiency (E.glob), modularity and small-worldness [i.e., σ (Humphries and Gurney, 2008) and ω (Telesford et al., 2011)], and the local-level attributes included nodal efficiency (E.nodal) and betweenness centrality (btwn.cent). A network was regarded as a small world when σ > 1 and when ω was within the range [−0.5 to 0.5]. Furthermore, we calculated the AUC for each topological attribute, which provides a summarized scalar for topological characterization of brain networks independent of single threshold selection.

### Statistical analysis

#### Comparative analyses

All comparisons in the present study were performed using general linear models (GLMs) with permutation tests. The statistical significance level was set at *P*_perm_ < 0.05. A network-based statistic (NBS) method was employed to determine intergroup differences in connectionwise connectivity strength (Zalesky et al., 2010). First, a GLM was specified for each element of the 246 × 246 connectivity matrix. A 246 × 246 matrix of *t*-statistics associated with the contrast of interest was thresholded by an initial *P* value threshold (*P* < 0.001). A graph was then created from this matrix, and the largest connected component was recorded. Next, the data were permuted 5,000 times according to the Freedman–Lane procedure in which each subject was randomly assigned to one of the subject groups. The same GLM was again specified at every matrix entry, a *t*-statistic matrix was calculated for the permuted dataset, and the associated *P* values were thresholded (*P* < 0.001). Finally, the largest connected component was recorded for the resultant graph, which was repeated for each permutation. The null distribution of the largest connected component sizes was used to calculate a *P*_perm_ value associated with the connected components of the observed data.

In terms of detecting intergroup differences in the AUC of topological attributes, the AUC data were permuted. The same GLM was tested on the permuted data, and the maximum statistic was recorded, building a null distribution. This procedure was repeated 10,000 times for global-level measures and 5,000 times for local-level measures. The null distribution of the maximum statistics and the observed statistics was compared to estimate a *P*_perm_ value for each attribute.

The topological attributes with significant intergroup differences were examined for Pearson’s partial correlations with clinical parameters (duration of MA use, age at first MA use, HAMA score, and BPRS and its five factor scores) controlling for age, education, and Fagerstrőm test for nicotine dependence (FTND) score. The correlations were adjusted for multiple comparisons using Bonferroni correction.

### Support vector machine-based classification

Least absolute shrinkage and selection operator (Zhang et al., 2021), a regularization and variable selection algorithm implemented in the glmnet package^4^ in R, was used to select an optimal subset of features from all global and nodal topological attributes with 10 repeats of fivefold cross-validation. The features selected were used to construct a linear SVM using the caret package^5^ in R. The procedure is similar to that detailed in our previous work (Li et al., 2019). In brief, a fivefold cross-validation framework was applied to evaluate the performance of the classifier. Before every cross-validation, the training dataset was scaled to between 0 and 1, and the acquired parameters were used to scale the test dataset. As the fivefold split is random, we repeated the fivefold cross-validation 100 times. The only parameter, C, which controls the trade-off between the margin width and the misclassification penalty, was set to the default value (*C* = 1).

Accuracy, sensitivity, specificity, and kappa were calculated to quantify the cross-validated predictive performance of these classifiers. Specifically, accuracy is related to the proportion of subjects who were correctly classified into the MA-dependence or HC groups, and sensitivity and specificity are related to the proportion of individuals in the MA-dependence and HC groups correctly classified. Kappa is similar to accuracy, except that it is normalized at the baseline of random chance on the dataset. The performance of the classifier was also assessed using receiver operating characteristic (ROC) curves from the results of the cross-validation data (Li et al., 2019). The area under the ROC curve (AUC) represents the classification power of a classifier, with larger AUC values indicating better classification power.

A one-tailed permutation test was utilized to evaluate the probability of obtaining cross-validation accuracy values higher than those achieved by chance. All subjects were randomly relabeled, and fivefold cross-validation classification was performed. This procedure was repeated 5,000 times, and the number of times the accuracy for the permuted labels was higher than that derived for the real labels was recorded. A *P*_perm_ value for the classification was then calculated by dividing this number by 5,000.

## Results

### Demographics and clinical data

The subject demographic features are displayed in Table 1. The subjects in the MA group had similar ages (34.43 ± 7.19), years of education (13.2 ± 4.2), and FTND scores (6.01 ± 2.78) to those in the HC group (age, 34.37 ± 10.31; education, 13.5 ± 3.7 years; and FTND, 5.98 ± 2.57). With respect to the MA group, the mean duration of self-reported MA use was 51.80 ± 36.99 months. The daily dosage of MA use was 0.45 ± 0.53 g. The age of the first MA use was 29.72 ± 7.20 years. The time between last MA use and MRI scan was 5.67 ± 1.08 days.

**TABLE 1:** Subject demographics.

	MA-dependent patients	HCs	*T*	*P*
Number	46	46		
Age (years)	34.43 ± 7.19	34.37 ± 10.31	0.035	0.972
Education (years)	13.2 ± 4.2	13.5 ± 3.7	−0.331	0.742
Duration of MA use (months)	51.80 ± 36.99	–	–	–
Daily dose of MA use (grams)	0.45 ± 0.53	–	–	–
Age of the first MA use (years)	29.72 ± 7.20	–	–	–
Time between last use and MRI scan (days)	5.67 ± 1.08	–	–	–
HAMA	22.39 ± 8.57	–	–	–
BPRS	39.11 ± 11.08	–	–	–
Anxiety-depression	12.8 ± 3.22	–	–	–
Lack of vitality	7.41 ± 2.9	–	–	–
Activity	6.5 ± 2.45	–	–	–
Hostility-suspicion	6.28 ± 3.01	–	–	–
Thinking disorder	6.09 ± 2.85	–	–	–
FTND	6.01 ± 2.78	5.98 ± 2.57	0.530	0.597

MA, methamphetamine; HC, healthy control; HAMA, Hamilton Anxiety Rating Scale; BPRS, Brief Psychiatric Rating Scale; FTND, Fagerstrõm test for nicotine dependence.

### Network-based statistic analyses

**FIGURE 1:**
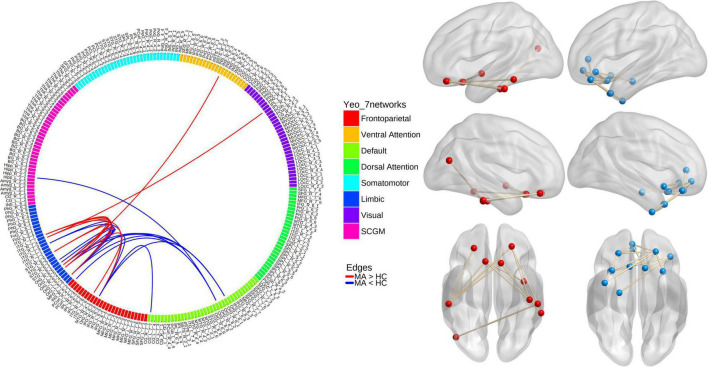
Results from network-based analyses of resting-state functional connectivity (rsFC) differences between methamphetamine (MA)-dependent patients and healthy controls (HCs). Red lines/balls: edges/nodes with increased rsFC; blue lines/balls: edges/nodes with decreased rsFC.

In the MA group, when compared with the HC group, the NBS analysis revealed one subnetwork with 10 nodes and 10 edges with an increased correlation (*P*_perm_ = 0.002) and another subnetwork with 12 nodes and 14 edges with a decreased correlation (*P*_perm_ < 0.001; Figure 1). For the 1st subnetwork, most increased connectivity existed between the orbitofrontal gyri (OFG) and inferior temporal cortices. Each decreased connectivity in the 2nd subnetwork had at least one node in the OFG, and half of these connections were present between subregions within the OFG. Based on the seven networks defined by Yeo et al. (2011), the brain regions that comprised the two subnetworks could generally be categorized into the following two groups of networks: ➀ the frontoparietal (FP) network, limbic network, visual network, ventral attention (VA) network, and subcortical gray matter (SCGM) network; ➁ the DMN, FP network, limbic network, and SCGM network.

### Graph theory analysis

**FIGURE 2:**
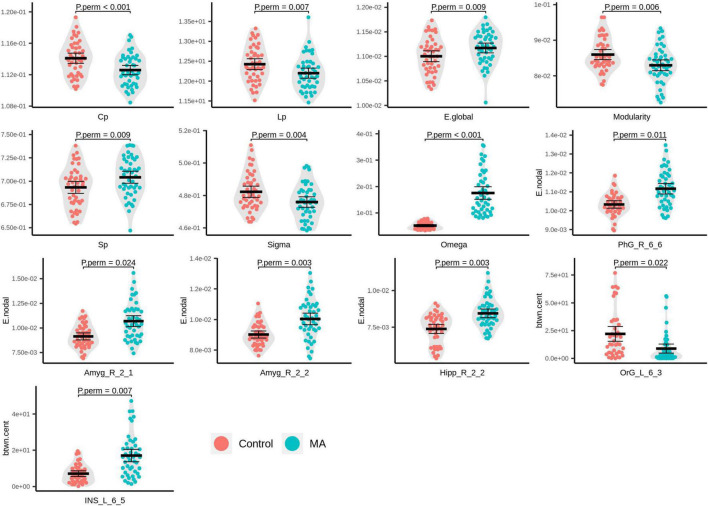
Beeswarm and violin plots of global/local topological attributes with evident inter-group differences. Red: healthy controls (HCs); green: methamphetamine (MA)-dependent group.

Compared with the HC group, the MA group demonstrated significantly decreased AUC for Cp (*P*_perm_ < 0.001), Lp (*P*_perm_ = 0.007), modularity (*P*_perm_ = 0.006), and small-worldness (σ, *P*_perm_ = 0.004), as well as increased AUC for E.glob (*P*_perm_ = 0.009), Sp (*P*_perm_ = 0.009), and small-worldness (ω, *P*_perm_ < 0.001), of the rsFC network (Figure 2). It should be noted that the closer ω is to 0, the more a network is considered small world. In the present study, the ω at every network sparsity threshold for each subject was smaller than zero, and the increased AUC for ω is suggestive of impaired small-worldness.

In the MA group, significantly increased E.nodal was detected in the right parahippocampal gyrus (PhG_R_6_6, *P*_perm_ = 0.011), right amygdala (Amyg_R_2_1, *P*_perm_ = 0.024; Amyg_R_2_2, *P*_perm_ = 0.003), and right caudal hippocampus (Hipp_R_2_2, *P*_perm_ = 0.003; Figure 2). With respect to btwn.cent values, the MA group manifested higher levels in the left dorsal granular insula (INS_L_6_5, *P*_perm_ = 0.007) and lower levels in the medial part of the left mOFG (OrG_L_6_3, *P*_perm_ = 0.022; Figure 2).

**FIGURE 3:**
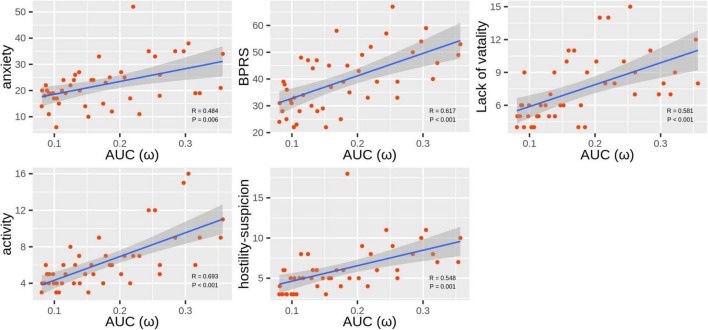
Scatter plots of correlations between ω and Hamilton Anxiety Rating Scale (HAMA) and Brief Psychiatric Rating Scale (BPRS) scores in the methamphetamine (MA)-dependent patients.

ω was the only topological attribute that presented significant associations with clinical variables: anxiety (*r* = 0.484, *P*_corrected_ = 0.006), BPRS scores (*r* = 0.617, *P*_corrected_ < 0.001), lack of vitality scores (*r* = 0.581, *P*_corrected_ < 0.001), activity scores (*r* = 0.693, *P*_corrected_ < 0.001), and hostility-suspicion scores (*r* = 0.548, *P*_corrected_ = 0.001; Figure 3).

### Support vector machine-based classification

From all 499 global and local topological attributes, 36 features were selected by LASSO, including small-worldness ω, E.nodal for 15 brain regions (INS_L_6_6, OrG_L_6_3, Tha_R_8_8, SPL_L_5_1, OrG_R_6_3, IPL_R_6_1, MTG_R_4_2, Hipp_R_2_2, BG_L_6_6, LOcC_R_2_2, Tha_L_8_3, PhG_R_6_6, Hipp_L_2_1, ITG_R_7_5, and Amyg_R_2_2) and btwn.cent for 20 brain regions (CG_R_7_1, SFG_R_7_5, IFG_L_6_1, OrG_L_6_4, INS_L_6_6, STG_L_6_5, OrG_L_6_3, STG_L_6_1, Amyg_R_2_1, pSTS_L_2_1, LOcC_R_4_1, IPL_R_6_4, BG_L_6_6, BG_R_6_6, SFG_L_7_2, Tha_L_8_3, IFG_L_6_4, STG_L_6_3, Hipp_R_2_2, and INS_L_6_5).

**FIGURE 4:**
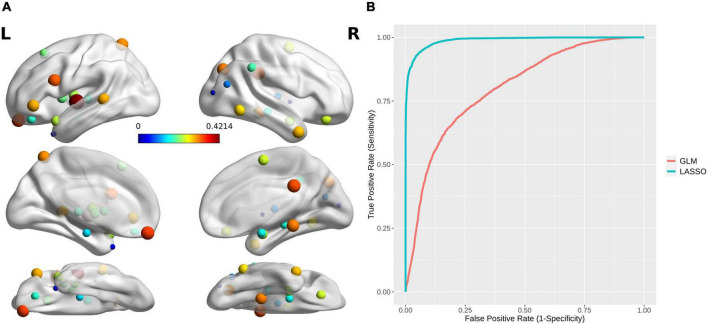
**(A)** The brain regions with topological attributes selected by Lasso for the construction of an SVM classifier for MA dependence. The size/color of each ball indicates the weight of every brain region with topological attributes. **(B)** Receiver operating characteristics curves for cross-validated prediction performance of classifiers trained on topological attributes selected using least absolute shrinkage and selection operator (LASSO) and general linear models (GLMs), respectively.

An SVM trained by the selected features exhibited excellent performance, with a cross-validated prediction area under the ROC curve, accuracy, sensitivity, specificity, and kappa of 99.03 ± 1.79, 94.00 ± 5.78, 93.46 ± 8.82, 94.52 ± 8.11, and 87.99 ± 11.57%, respectively (*P*_perm_ < 0.001) (for the detailed weights of the 36 features in the SVM model, see Table 2 and Figure 4A; for the ROC for cross-validated prediction performance of classifiers trained on topological attributes selected using LASSO, see Figure 4B).

**TABLE 2:** The weights (absolute values) of the 36 features in the support vector machine (SVM) model selected by least absolute shrinkage and selection operator (LASSO).

Topological attributes	Brain region	Weight
btwn.cent	STG_L_6_3	0.421360994
btwn.cent	OrG_L_6_4	0.342771471
btwn.cent	CG_R_7_1	0.335849235
E.nodal	IFG_L_6_1	0.327454015
E.nodal	LOcC_R_2_2	0.312574091
E.nodal	PhG_R_6_6	0.311454086
E.nodal	SPL_L_5_1	0.310386064
btwn.cent	pSTS_L_2_1	0.294406751
btwn.cent	IFG_L_6_4	0.294347901
E.nodal	MTG_R_4_2	0.287266929
E.nodal	ITG_R_7_5	0.272386256
btwn.cent	SFG_R_7_5	0.243216991
E.nodal	OrG_R_6_3	0.239476562
btwn.cent	STG_L_6_5	0.233816459
E.nodal	Amyg_R_2_2	0.229682465
btwn.cent	INS_L_6_5	0.22587306
btwn.cent	SFG_L_7_2	0.209940876
E.nodal	BG_L_6_6	0.205896391
btwn.cent	INS_L_6_6	0.200320952
btwn.cent	IPL_R_6_4	0.187731758
btwn.cent	Tha_L_8_3	0.175864611
btwn.cent	OrG_L_6_3	0.172619151
E.nodal	Hipp_R_2_2	0.165738702
btwn.cent	Amyg_R_2_1	0.162644422
ω		0.158284882
E.nodal	INS_L_6_6	0.153673004
E.nodal	OrG_L_6_3	0.152450515
E.nodal	Hipp_L_2_1	0.14171235
E.nodal	Tha_L_8_3	0.134457447
E.nodal	Tha_R_8_8	0.115415487
E.nodal	IPL_R_6_1	0.100856487
btwn.cent	STG_L_6_1	0.048967981
btwn.cent	BG_L_6_6	0.033907512
btwn.cent	LOcC_R_4_1	0.03328945
btwn.cent	Hipp_R_2_2	0.015441907
btwn.cent	BG_R_6_6	0.004633169

btwn.cent, betweenness centrality; E.nodal, nodal efficiency.

## Discussion

To our knowledge, this is the first study to analyze rsFC using Tikhonov regularization, and investigate altered AUC of rsFC-based topological attributes in SUDs. In the present study, NBS analysis on Tikhonov regularization-based rsFC data revealed a subnetwork with increased FC and a subnetwork with decreased FC in MA-dependent patients. The brain regions that comprised the two subnetworks originated from the FP, limbic, visual, VA, and SCGM networks, and most of these brain regions were located in the OFG. By using graph theory on these data, the MA-dependent patients presented altered AUC of global topological attributes, i.e., decreased small-worldness (σ, ω), Cp, Lp, and modularity and increased E.glob and Sp. ω was the only topological attribute that presented significant correlation with HAMA/BPRS scores in the MA group. Moreover, aberrant local topological attributes, i.e., three regions with increased AUC of E.nodal, one region with increased AUC of btwn.cent and one region with decreased AUC of btwn.cent were also discovered in the patients. An SVM trained with features selected by LASSO from AUC of all topological attributes achieved excellent classification of MA-dependent patients with a cross-validated accuracy of 94.00 ± 5.78%.

### Intergroup differences in resting-state functional connectivity

Confirmed by diffusion tractography imaging (Hsu et al., 2020), the orbitofrontal cortex (OFC) receives visual inputs directly from the inferior temporal cortex (ITC) and then projects back to the ITC (Rolls, 2000). The responses of the visual neurons in the OFC encode the reward value of visual stimuli. As most increased FC values in the MA-dependent patients existed between the OFC and ITC in this study, it suggests enhanced transmission of information between the two structures and may indicate overactivation of the reward circuit, making patients very sensitive to drug-related visual cues.

As a functionally heterogeneous structure, the OFC can be divided into multiple regions distinguished by cytoarchitecture with strong fiber connections between subregions (Hsu et al., 2020). This structure has been proven to play a critical role in compulsive drug-seeking and drug relapse (Schoenbaum and Shaham, 2008). A number of MRI and metabolic studies have reported altered structure (Daumann et al., 2011; Nakama et al., 2011; Li et al., 2017) and hypofunctionality (Volkow et al., 2001; Paulus et al., 2002, 2003; Sekine et al., 2003; London et al., 2004; Li et al., 2019) of the OFC in MA-dependent patients. However, very few studies have explored FC patterns within the OFC in patients with SUDs. The NBS analysis in this study revealed that in the MA-dependent subjects, all associations with decreased connectivity had one node in the OFG, and half of these connections were with subregions within the OFC. Similarly, by using voxel-mirrored homotopic connectivity (VMHC), an index to measure the strength of the functional connection between a voxel/ROI and its counterpart in the opposite hemisphere, researchers have found significantly reduced VMHC between the bilateral medial OFCs (mOFCs) in patients with internet gaming disorder (Chen et al., 2020) or codeine cough syrup-dependence (Qiu et al., 2017). Ieong and Yuan (2017), by using functional near-infrared spectroscopy in heroin users, also reported weaker functional connections between these structures. These results confirmed the key role of the OFC in the development of MA dependence and indicate widespread MA-related impairment in synchronized spontaneous activity of subregions within the OFC system, which are thought to be implicated in the core characteristics of SUDs, such as biased associations between stimuli and reward responses and deficits in properly inhibiting drug intake and aversive/withdrawal reactions to potentially dangerous situations (Goldstein et al., 2005). However, in addition to a decreased connection between the bilateral lateral OFC, Ma et al. (2010) found stronger rsFC between the left OFC (left lOFC) and right mOFC and between the bilateral mOFC in heroin users. These discrepant results may have stemmed from different data processing methods and different drugs used and require further investigation to uncover the neuromechanisms underlying the altered rsFC circuits within the OFC system.

In this study, connectivity between the right subgenual anterior cingulate gyrus (sgACC, CG_R_7_7) and the right medial OFG (right mORG, ORG_R_6_3, BA 11) was significantly attenuated in MA-dependent patients. Both of these structures have been verified to play important roles in regulating emotion (Rudebeck et al., 2014). Ramirez-Mahaluf et al. (2018), by applying graph analysis methods to rsFC data, identified a brain network that included the sgACC and mOFG and is associated with sadness processing. Moreover, the stronger the rsFC between these two structures is, the better the clinical response to accelerated intermittent theta burst stimulation (a repetitive transcranial magnetic stimulation protocol) in patients with depression (Baeken et al., 2017). As there is a high prevalence of comorbid anxiety/depression and MA dependence (National Institutes on Drug Abuse, 2020), this disrupted connectivity was presumed to be a brain imaging biomarker for mood disturbance in MA-dependent individuals. London et al. (2004), by using [^18^F]fluorodeoxyglucose positron emission tomography, revealed MA-related significantly lowered glucose metabolism in the sgACC, which may underlie this weakened connectivity.

By using task-fMRI-based FC analysis, Ross et al. (2013) proved the critical role of connectivity between the lOFC and hippocampus in both working memory and long-term memory when separate representations of overlapping stimuli need to be disambiguated, which is important for social interaction, i.e., enabling us to distinguish between changing contexts and social situations and then to act appropriately. The same connectivity under resting-state conditions, i.e., rsFC between the lateral part of the left OFG (left lOFG, OrG_L_6_6) and the hippocampus (Hipp_R_2_1), was weakened in MA-dependent patients in the current study. Related to these results, MA-induced long-lasting impairments in working memory have been reported in male rats (Braren et al., 2014). Several studies have also yielded a correlation between impaired working memory and MA dependence in both current and abstinent chronic MA users (Guerin et al., 2019). We postulate that this altered connectivity may indicate impaired working memory in patients, which needs further research by analyzing the correlation between this rsFC and measures of working memory.

### Intergroup differences in global topological organization in the resting-state functional connectivity network

A healthy human brain is a typical model of a small-world network. The characteristics of small-world networks, i.e., high Cp and short Lp with optimized balance between functional segregation and integration, make it possible to maintain efficient and specialized modular information processing and rapid global information transmission in the network (Rubinov and Sporns, 2010). In the present study, although the nature of small-world architecture was conserved in the MA group, the aberrant AUC for σ and ω in this group indicated impaired small-worldness that, together with decreased Cp (a measure of network functional segregation), Lp (a measure of network integration), and Sp (a measure of network density or the total “wiring cost” of the network) and increased E.glob imply an MA-related shift toward random networks (Jiang et al., 2013; Zhu et al., 2016; Park et al., 2017). The shift in brain functional network configuration toward a random network organization has been exhibited for internet gaming addiction (Park et al., 2017), heroin dependence (Jiang et al., 2013), and other neuropsychiatric disorders, such as schizophrenia (Zhu et al., 2016), depression (Long et al., 2015), and Alzheimer’s disease (AD) (Stam et al., 2009). These kinds of topological alterations suggest less functional segregation, which also lead to a less optimized functional organization, in the MA-dependent brain.

In addition, the MA group in this study demonstrated lower modularity, a global topological attribute measuring the division of a network into separate modules (Rubinov and Sporns, 2010). A module is characterized as having denser connections between nodes within the module but sparser connections with nodes outside the module. Reduced network organization, implied by lower modularity and Cp in MA-dependent brains, suggested poor functional network segregation. Consistent with these results, single-cell whole-brain imaging-based functional networks from male mice administered cocaine/MA/nicotine for 1 week (Kimbrough et al., 2021), as well as white matter structural networks in synthetic cannabinoid users (Celik et al., 2020), have also presented decreased modularity. By using simple computer simulations on the dynamics of modularization in a minimal substrate, Lipson et al. (2002) suggested that modularity can spontaneously arise under changing environments, i.e., higher modularity results in higher adaptability of their behavior to acquire rewards and to avoid punishments (Hu, 2016). As impairments in this ability are one of the main symptoms of addiction, we presume that decreased modularity in MA dependence is closely related to the failure to adapt to environmental changes, which then results in aberrant reward function and behavior.

### Intergroup differences in local topological attributes in the resting-state functional connectivity network

In the patients in this study, the brain regions with significantly increased E.nodal included the right amygdala and hippocampus, and a similar pattern was also discovered in patients with major depressive disorder (Ye et al., 2016). The hippocampus and amygdala are core parts of the affective processing network. Specifically, the amygdala manifests greater activity that lasts longer when depressed patients process negative stimuli, and the hippocampus shows enhanced activity in the recall of negative, not positive, stimuli after encoding in the amygdala (Ye et al., 2016). Therefore, it is plausible that these results provide biomarkers for comorbid depression and MA dependence from the perspective of the local topology.

The insular cortex is known to be involved in interoception, decision making, anxiety, pain perception, cognition, mood, threat recognition, and conscious urges (Ibrahim et al., 2019). In MA users, the left insula has been reported to present volume reduction (Hall et al., 2015), greater activation across risky and safe decisions (Gowin et al., 2014), and decreased glucose metabolism (Vuletic et al., 2018). Consistent with these findings, our results, i.e., the left insula as the only structure with MA-related increment of betweenness centrality and as important features for constructing a high-efficacy classifier, supports the perspective that left insular cortex is involved in key aspects underlying MA-dependence.

It has been suggested that the mOFC is involved in processing stimulus-reward associations and with the reinforcement of behavior (Rolls, 2000). Moreover, in a task-fMRI-based FC and rsFC study on alcohol use disorder that enrolled 1890 subjects, the mOFG was suggested to be a central/core structure in a neural network that may underlie the onset of alcoholism (Jia et al., 2021). In this network, the authors identified two independent regulatory pathways between the mOFC and the dorsal periaqueductal gray (dPAG) in the brainstem contributing to alcohol abuse; in particular, excitatory regulation during the resting state was related to impulsive behavior, and inhibitory regulation upon receiving a non-reward (relative punishment) was related to compulsive alcohol use. In the present study, the left mOFC with a significant reduction in betweenness centrality, which is a more sensitive measure of the importance of a node connecting disparate parts of a network, may indicate dysregulation of this core structure in the substance dependence-related network and hence inevitably lead to dependence on MA.

### Support vector machine classifier

The only global topological attribute selected by LASSO to train an SVM was small-worldness ω. The currently accepted definition of a small-world network is that it has clustering comparable to a regular lattice and Lp comparable to a random network. σ is defined by comparing clustering and Lp to that of a comparable random network, which means that networks with very low clustering can also be, and indeed are, defined as small world. To solve this defect, Telesford et al. (2011) introduced, which is defined by comparing network clustering to an equivalent lattice network and Lp to a random network; ω has the following advantages: (a) it provides a more accurate estimation of small-worldness; (b) it is less sensitive to the size of a network and benefits from inherent scaling, which provides a powerful tool for comparing and ranking small-world properties in various systems. From the perspective of machine learning, our results suggested that ω is a better feature for the classification of MA dependence than the other global topological attributes. Moreover, the evident positive associations between the AUC for ω and HAMA/BPRS scores in the MA group indicate that ω may be an ideal brain network marker for the psychiatric symptoms of MA-dependent patients.

Most of the regions with local topological attributes selected by LASSO were from the DMN, limbic, FP, visual, attention, and SCCM networks, covering four addiction-related interacting circuits (reward, motivation/drive, memory and learning, and control), attention circuits and emotion circuits as proposed by Volkow et al. (2003). The excellent classification accuracy of the SVM in the present study indicates that topological attributes derived from rsFC networks may be promising features for constructing a high-efficacy classifier for MA-dependent individuals.

### Limitations

Several considerations should be taken into account with respect to the interpretation of the present results. First, the relatively small sample size made it difficult to further analyze the differences in rsFC networks between subgroups (e.g., between male and female patients, between patients without psychosis and, those with psychosis). Moreover, a potential overfitting problem for the classifier can hardly be avoided without establishing an independent test dataset. A larger sample size in future studies would help enhance the generalizability of the present classifier. Second, datasets with other SUDs are needed to tell whether the methods applied in the present study could also be sensitive to discover altered rsFC-based connectome, and could construct a high-efficacy classifier using rsFC-based topological attributes, in patients with other SUDs. Third, the low sampling rate (TR = 2 s) in the present study cannot completely eliminate the effects of physiological noise, such as cardiac and respiratory noise, on BOLD signals, which can affect the calculation of rsFC, especially within the DMN, even after the application of bandpass filtering (Yoshikawa et al., 2020). In future studies, a faster sampling rate or algorithm for removing physiological noise should be applied to address this problem (Liao et al., 2013; Yoshikawa et al., 2020). Fourth, as this is a cross-sectional study and no correlations were found between the topological attributes and duration of MA use/age at first MA use, it is unclear whether the altered topological attributes were the result of MA dependence or a pre-addiction condition, which could be clarified by genetic and longitudinal imaging studies in the future.

In conclusion, the present study demonstrates an abnormal brain Tikhonov regularization-based rsFC connectome in MA-dependent patients from the perspectives of FC and topology. The MA-related alterations in brain rsFC networks present a shift toward random networks. The rsFC-based topological attributes may be promising features for constructing high-efficacy machine-learning-based classifiers for MA-dependent individuals, which needs verification from a larger sample size.

## Data availability statement

The raw data supporting the conclusions of this article will be made available by the authors, without undue reservation.

## Ethics statement

The studies involving human participants were reviewed and approved by the Institutional Review Board of Ningbo Medical Center Lihuili Hospital, Ningbo University, Zhejiang, China. The patients/participants provided their written informed consent to participate in this study.

## Author contributions

YL and WZ were responsible for the study concept. YL conducted the literature searches, data analyses, and drafted the manuscript. PC and HD were provided expertise in statistical analyses and multivariate pattern classification. LL performed the MRI data collection. HL and WS were responsible for recruitment, interview of all subjects, and collection of their clinical data. WS provided diagnosis for all patients. WZ provided critical revisions of the manuscript. All authors reviewed the manuscript and approved the final version for publication.

## Footnotes

1
https://www.unodc.org/unodc/en/data-and-analysis/wdr2021.html

2
http://www.nncc626.com/2022-06/23/c_1211659746.htm

3
https://github.com/cwatson/brainGraph

4
https://cloud.r-project.org/package=glmnet

5
https://github.com/topepo/caret/
